# Gendered Risk Perceptions Associated with Human-Wildlife Conflict: Implications for Participatory Conservation

**DOI:** 10.1371/journal.pone.0032901

**Published:** 2012-03-05

**Authors:** Meredith L. Gore, Jessica S. Kahler

**Affiliations:** 1 Department of Fisheries and Wildlife, School of Criminal Justice, Michigan State University, East Lansing, Michigan, United States of America; 2 Department of Fisheries and Wildlife, Michigan State University, East Lansing, Michigan, United States of America; University of Pretoria, South Africa

## Abstract

This research aims to foster discourse about the extent to which gender is important to consider within the context of participatory approaches for biological conservation. Our objectives are to: (1) gender-disaggregate data about stakeholders' risk perceptions associated with human-wildlife conflict (HWC) in a participatory conservation context, and (2) highlight insights from characterizing gendered similarities and differences in the way people think about HWC-related risks. Two communal conservancies in Caprivi, Namibia served as case study sites. We analyzed data from focus groups (*n* = 2) to create gendered concept maps about risks to wildlife and livelihoods and any associations of those risks with HWC, and semi-structured interviews (*n* = 76; men = 38, women = 38) to measure explicit risk attitudes associated with HWC. Concept maps indicated some divergent perceptions in how groups characterized risks to wildlife and livelihoods; however, not only were identified risks to wildlife (e.g., pollution, hunting) dissimilar in some instances, descriptions of risks varied as well. Study groups reported similar risk perceptions associated with HWC with the exception of worry associated with HWC effects on local livelihoods. Gendered differences in risk perceptions may signal different priorities or incentives to participate in efforts to resolve HWC-related risks. Thus, although shared goals and interests may seem to be an obvious reason for cooperative wildlife management, it is not always obvious that management goals are shared. Opportunity exists to move beyond thinking about gender as an explanatory variable for understanding how different groups think about participating in conservation activities.

## Introduction

Not all human-wildlife interactions result in conflict, however when they do occur, human-wildlife conflicts (HWC) (i.e., interactions that result in negative effects for people or wildlife) can pose risks to wildlife conservation and livelihood preservation [1]. HWC may range in both magnitude (e.g., death, injury, property damage, vulnerability, political conflict [2]–[3]) and frequency (e.g., stochastic, seasonal, year round incidents [4]). Around the world HWC can pose severe problems for people such as decreased food security, increased workload, decreased physical and psychological well being, economic hardship, and at times an increase in illegal or dangerous activities such as poaching [5]. Debate among affected parties regarding appropriate management responses to HWC can erupt [6] and may generate political conflict between people and institutions (see Treves et al. [6] for additional information about types of interventions used to resolve conflicts) [7]. HWC can be similarly problematic for wildlife, contributing to population suppression, range collapse, or extinction [8]. HWC is a highly complex phenomenon that transcends ecological, economic, management, political, and social systems [1].

Resolving HWC fundamentally requires managing risk [3], [9]. Solutions are often forged on community-based or participatory approaches [10]–[11], particularly in regions of the world where management agency resources are limited or decentralized. Such efforts ideally incorporate context-specific factors [12]–[13]. Participatory approaches to biodiversity conservation can be viewed differently by various subgroups of people within a community [5], [14]–[15]. Who has a voice in community conservation influences how well a group functions and who gains and loses from or is affected by interventions [14]. Neglecting stakeholders can lead to an incorrect assessment of intervention success in terms of achieved levels of equitable participation and efficiency. This may result in devastating and irreversible impacts for wildlife and people [1]. Omitting stakeholders may also obscure the difference between those who have a stake in [wildlife conservation] and those who have the ability to act on it [14]. Participatory approaches often aim to overcome stakeholder neglect by purposefully including diverse stakeholders in wildlife decision-making. Ideally the approach leads to more democratic, executable, and creative management decisions through increased diversity in issue-related information and perspectives on conservation issues [16]–[17].

Although we may expect differences among different stakeholders' perceptions of and preferences for participatory HWC management [18], differences are not always purposefully measured or incorporated (e.g., Agrawal [10], Lauber et al. [19], Anthony et al. [18], Gore et al. [20], Ogra [5]). Whereas historically wildlife decision-making literature focused primarily on stakeholder groups who were mostly comprised of men (e.g., see Anthony et al. [18]), women are now recognized as important players in contemporary conservation contexts [18]. Given the potential for gender differences in wildlife-related attitudes, perceptions, and behaviors, women need to be recognized as a unique and critical stakeholder group in HWC-related decisions [18], [21]. In some instances, policies are already in place to advance this objective. For example, Millennium Development Goal Three (i.e., promote gender equality and empower women) explicitly focuses on gendered dimensions of education, poverty, politics, and vulnerability [22]. Ideally, engaging women in decision making over the use and management of local environmental resources is central not only to women's empowerment but also to greater sustainable development [16], [17], [23].

Gendered risk perception is currently an understudied dimension of biodiversity conservation and HWC, but does gender warrant explicit consideration by HWC scholars and practitioners? Is thinking about gendered participation in HWC interventions necessary or sufficient for conservation project success? What are the gendered costs and benefits of participating in HWC interventions? Gender scholars (e.g., Ray [23]) have purported answers to these questions are tentative at best because some environmental resource sectors, including biodiversity conservation, are weak in the kinds of data that are needed to arrive at definite answers. Our goal is to foster discourse about the extent to which gender is an important dimension to consider within the context of participatory approaches to biological conservation, such as resolving HWC-related risks. Even though gender is often used synonymously with women in environmental contexts, including biodiversity conservation, we follow Nightingale's [24] approach and focus our gendered inquiry on both men and women.

### Gendered perceptions in conservation

Political ecologists often consider gender an explanatory factor for variance in attitudes about conservation issues such as fishing policy [25], perceptions of a biosphere reserve [26], participation in conservation programs [27], and predictors of wildlife value orientations [28]. Scholars have explored gender differences in fishing acculturation [29], citizen participation [18], fairness in wildlife decision- making [19], and considered gender bias in U.S.-based wildlife survey research [30]. Men are more likely to be accepting of hunting whereas women are more likely to express anti-hunting attitudes [31]. Women often perceive greater risk from contact with wildlife [31]–[32] and have demonstrated greater concern for the impacts of wildlife conservation and management on their local communities [19]. Scholars have also explored gendered effects on attitudes associated with HWC (e.g., Ogra [5]).

Considering gender differences in attitudes towards wildlife and wildlife conservation has given practitioners a richer understanding of the concerns different stakeholders bring to the table [19]. For example, Gilligan [33] discussed how men commonly exhibit an ethic of justice (e.g., rules about actions being right or wrong) and women an ethic of care (e.g., actions that preserve and nurture relationships) when thinking about policy preferences for natural resource management. Lauber et al. [19] found men and women used different criteria to evaluate wildlife conservation alternatives. Gender differences can be detected in wide-ranging topics such as support for conservation schemes, willingness to participate in cooperative management solutions, or attribution of responsibility for resolving HWC [34]. Further, gender differences in the types of criteria used to evaluate conservation alternatives can also explain conservation preferences. Women may emphasize some criteria more than men and have different preference levels for the relative importance of such criteria [19]. We acknowledge many of these conclusions are based on research conducted with Western cultures and there are likely differences between these Western-based studies and an African context. However, it is reasonable that similar considerations are important in non-Western cultures and conservation contexts [34].

### Risk perceptions and gender

Risk perception (i.e., intuitive judgments as opposed to technical assessments about risk [35]) has been applied to gender and HWC (see Gore et al. [36] for a broad review of risk and wildlife, including psychological factors influencing wildlife-related risk perception), although the three concepts are rarely, if ever, applied together. The risk and decision sciences literature tells us men and women commonly differ in their perception of risk [37]–[38]. Variations in risk perceptions seem to reflect not only gender differences in activities and social roles, but also unequal power relations and different levels of trust in authorities and institutions [39].

Psychologists offer insights about perceptual differences between risks [36]. As with the wildlife-related literature above, many psychological studies have focused on individuals from Western cultures and thus reference types of risk relevant to the study population (e.g., nanotechnology in Europe). Regardless of the risk topic or society studied, gender differences persist. Thus, we know men tend to express less concern for many *types* of risks such as climate change or sexual assault [38]. Some risk perception studies reflect predominantly male experiences [40] and present women as an out-group comparison to the male in-group. One example is the proposition that women repeatedly prioritize home and family, mainly perceive risks as threats to their family and other persons with whom they had close relations, and to their home (e.g., fire) [38].

The influence of gender on risk perception is dynamic and complex. Women and men may perceive the same risks differently, they may perceive different risks, and they may attach different meanings to what appear to be the same risks [38]. Thus, it is important not only to describe but to also explain gender differences in risk perception. We suspected, similar to Ogra [5], that many important dimensions of HWC, such as risk perception, go unnoticed in part because they are gendered. To this end, we set the following objectives, to: (1) gender-disaggregate data about stakeholders' risk perceptions about risk to wildlife, livelihoods, and any associations of those risks with HWC in a participatory wildlife conservation context, and (2) highlight the important insights that arise in characterizing gendered similarities and differences in the way people think about risks associated with wildlife, livelihoods, and HWC.

## Methods

### Ethics statement

The methods for this research were approved for the duration of the project by the MSU Committee on Human Subjects, Protocol ID # X09-443. A Committee-approved verbal informed consent procedure was used due to potential participant illiteracy. In instances where participants approved use of a digital voice recorder, consent was documented digitally. In all instances, participants had to verbally consent to participating in the study before data collection commenced.

### Study area

The Caprivi region of northeastern Namibia is a narrow swath of land covering approximately 20,000 km^2^ and bordering Angola, Zambia, and Botswana. The region has four major rivers, the highest rainfall in Namibia [41], and is home to approximately 80,000 people [42]. Local livelihoods in Caprivi are highly dependent on natural resources [42]. Caprivi has high concentrations of wildlife including one of the largest populations of free-ranging elephants (*Loxodonta africana*) in Africa [41] and a diverse assemblage of predators including lion (*Panthera leo*), spotted hyena (*Crocuta crocuta*), leopard (*Panthera pardus*), and crocodile (*Crocodylus niloticus*). Resolving HWC in Caprivi is a priority because it causes an estimated annual economic loss of USD $770,000 [42] through livestock depredation and damage to commercial crops; this number does not include injury, death or damage to non-commercial crops, all of which occur. Within Caprivi we selected communal conservancies (i.e., decentralized, communal community-based management units in Namibia) for this research (Table 1) based on the following criteria: (1) willingness to participate, (2) documented presence of HWC, and (3) the second author being granted verbal permission to conduct research by traditional authorities and conservancy managers.

**Table 1:** Environmental and socio-demographic characteristics of study site and interview participants (*n* = 76) in East Caprivi, Namibia [64].

**Environmental characteristics**	
Adjacent conservation areas	Balyerwa Communal Conservancy (west); Mamili National Park (south); Mudumu National Park (north); Shikakhu Community Forest (east)
Climate	Semi-arid (Average annual rainfall ≤625 mm)
Major wildlife resources^a^	Buffalo, Duiker (*Cephalophus sp.*), Elephant, Impala (*Aepyeros melampus*), Kudu, Leopard, Lion, Reedbuck (*Redunca arundinum*), Roan (*Hippotragus equinus*), Tsessebe (*Damaliscus lunatus*), Warthog (*Phacochoerus africanus*), Wildebeest (*Connochaetes taurinus*)
Size	393 km^2^
Terrestrial ecoregion classification	Mosaic of Zambezian *Baikiaea* woodlands and flooded grasslands
**Socio-demographic characteristics of interview participants (n = 76)**	
Approximate population (density); average household size (range)	2,491 (6.34/km^2^); 5.83 (1–15 people)
Ethnic group composition	Mayeyi (94%); Totela (4%); Mafwe (1%); Kwanyama (1%)
Educational attainment	No school (17%); some primary (16%); completed primary (3%); some secondary (42%); completed secondary (18%); some college (1%); adult vocational (3%)
Participation in livelihood strategies	Agriculture (97%); rural industry (26%); livestock (25%); fishing (16%); business (8%); commercial farming (3%); NGO (3%); tourism (3%); government (1%); other (3%)
Local languages	Sheyeyi (83%); Lozi (13%); Subiya (2%); Totela (2%)
Traditional authorities	Chief Mbambo; Chief Sifu

### Data collection

We used focus groups and semi-structured interviews [43] to achieve objectives. Focus group participants were solicited from each village zone (i.e., distinct residential area) within two Caprivi conservancies (i.e., Wuparu, Dzoti). Participants were solicited using a cluster and convenience sampling technique with probability proportionate to size [43]. Interview participants were recruited from the same conservancies. All study participants were permanent residents of their respective conservancies and 18 years of age or older. Participation in one research activity did not exclude participation in the other. However, interview and focus group participants were independently selected using the above-described sampling protocols to maximize participation and minimize burdens on participants (e.g., time away from work). Six translators, five men and one woman, were trained by the second author and certified according to MSU Institutional Review Board requirements. Translators: (1) were fluent in English, Lozi, and/or Sheyeyi, (2) had completed secondary school, (3) were not members of the traditional authority, and (4) agreed to work the duration of research activities. The second author relayed these job requirements to conservancy staff who then made recommendations on possible applicants; applicants were then interviewed, hired, and trained. Data collection instruments were pretested to increase the validity of instrument content [45].

The second author and six translators facilitated identical two-day focus groups in each conservancy (see Kahler [45] for protocol). Participants were divided into three parallel groups comprised of: (1) male residents, (2) female residents, and (3) local environmental decision makers of any gender to promote a nonthreatening and permissive environment for dialogue [46] and help diffuse potential power differentials between participants [47]. During the focus group activity participants first individually free-listed risks associated with two broad risk targets (i.e., wildlife, local livelihoods) on an index card so as not to constrain responses to researcher-imposed ideas about HWC-related risks. Within groups, participants shared and discussed their results and identified what, if any, risks from the two targets were related to HWC. The activity was completed independently for the two risk targets. At the conclusion of each focus group the second author and translators reviewed index cards. Risks not listed or defined in English were translated by consensus to maximize the reliability and validity of translation for future analysis.

Semi-structured interviews commenced concurrently with focus groups and measured participant demographic characteristics (e.g., age, education) as well as explicit attitudes about risks to wildlife and livelihoods associated with HWC, such as perceptions of risk to wildlife from HWC, factors influencing risk perceptions associated with HWC such as dread or worry, compliance with wildlife rules, and vulnerability associated with HWC (see Kahler [45] for interview instrument). Questions were queried using four-point visual Likert-type scales (see Kahler [45] for scales) to lessen the potential for culturally driven bias towards neutral or extreme response categories [48]–[49]. Such scales are also appropriate in situations of low literacy [50].

### Data analysis

An iterative process guided the first phase of our qualitative, gendered analysis of focus group data [20], [51]. We used this type of analysis because the process does not dilute participant comments and data is minimally constrained by the researcher [52]. Because we had two risk targets (i.e., wildlife, local livelihoods), we conducted separate coding iterations for both. First, we reviewed all participants' livelihood risk lists and identified “overarching risk themes” across all livelihood risks (e.g., lack of access to education). These themes informed the next phase of analysis, where we reviewed participants' “overarching risk theme” lists to identify “theme attributes” (e.g., lack of family planning) and “descriptions of the attributes” (e.g., increased population). We conducted a third iteration of coding to recheck our work [44]. After identifying “overarching risk themes,” “attributes,” and “descriptions” for livelihoods and wildlife across all participants as noted above, we gender disaggregated the data using LeCompte and Goetz's [53] methodology whereby we scanned, ordered, reviewed, and compared concepts. We used concept mapping, or a visual display illustrating relationships between and among concepts [52], to visually delineate gendered perspectives of risks to wildlife and livelihoods and those risks associated with HWC. Concept mapping is useful for summarizing the ideas of a group without losing individuality, trivializing some ideas over others, or losing detail [44]. We generated six concept maps, three each for livelihoods and three for wildlife. Each concept map was anchored upon one of the three dimensions commonly associated with HWC, “people,” “wildlife,” and “habitat” [54]. Tethered to each dimension were the “overarching risk themes,” “theme attributes” and “descriptions” participants identified during focus groups. Finally, we visually compared gendered concept maps to identify concepts in common to men and women (delineated with bold-faced type in figures) as well as concepts the participants delineated as related to HWC (shown in italicized type in figures).

Responses to interview questions were recoded into dichotomous variables for quantitative analysis. Variables were cross-tabulated to assess the percentage of positive responses among the sample and chi-square tests for independence were calculated using PASWStatistics 18.0 [55].

## Results

### Focus groups

Focus group results illustrate the diversity of risks facing men and women in the study area. Focus group participants generated 315 risk index cards: thirty-two participants (men = 15, women = 17) generated 151 index cards for wildlife as the risk target and 33 participants (men = 16, women = 17) generated 164 index cards for livelihoods as the risk target. Women generated a list of 81 risks for the wildlife target and 99 risks for the livelihood target. Men generated a list of 70 risks for the wildlife target and 65 risks for the livelihood target. Female participants ranged in age from 18 to 63 years old, three were illiterate in either their native language or languages taught in school (i.e., English, Lozi) and their formal education background ranged from never having attended school to post-secondary vocational and technical training. Male participants ranged in age from 18 to 46 years old, all were literate in at least their native language and their formal education background ranged from having completed some primary school to having completed a college degree.

We explored gendered perceptions about risks to wildlife and livelihoods and those risks associated with HWC using concept maps. Concept maps illustrated both similarities and difference in participant impressions. We discuss these impressions below.

### Participants' livelihood concept maps

**Figure 1:**
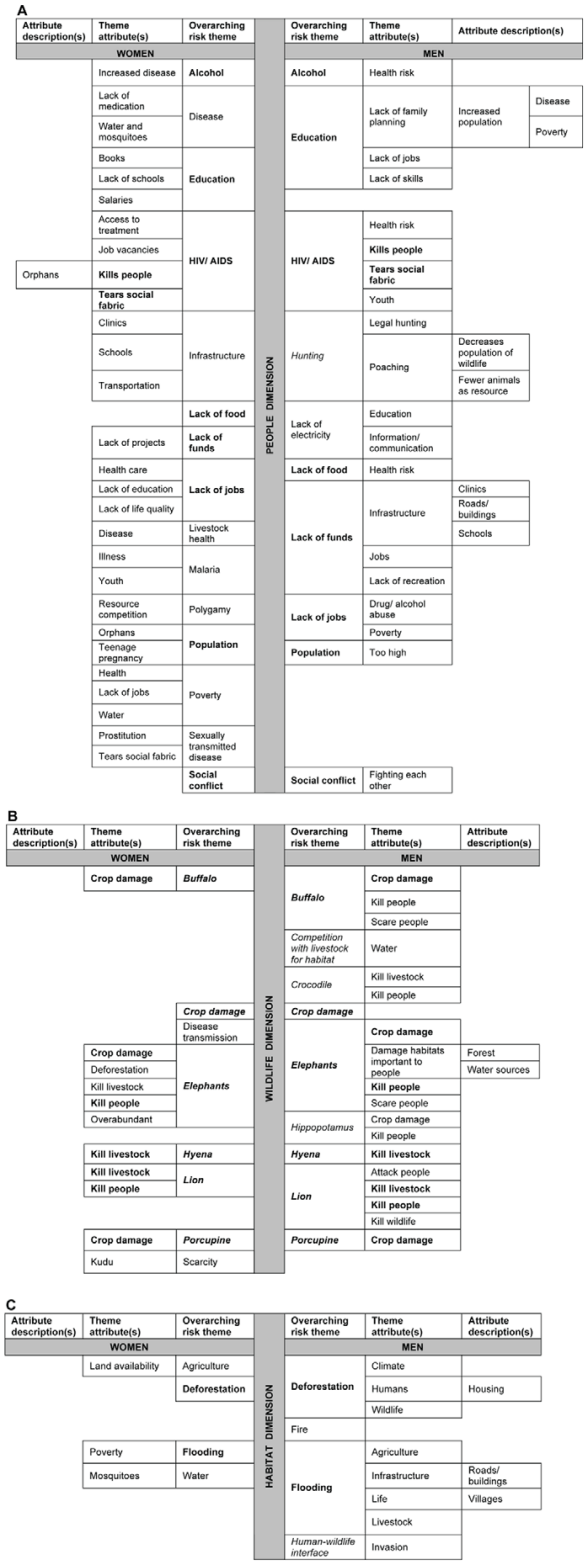
Concept maps illustrating participants' perceptions of risk to livelihoods. Focus group participants (n = 33; men = 16; women = 17) generated a list of risks to their livelihoods and described them. Responses are displayed in a gender-segregated concept map presenting the “overarching risk themes,” “theme attributes,” and “attribute descriptions.” Fig. 1A displays responses anchored in the “people dimension,” Fig. 1B displays responses anchored in the “wildlife dimension,” and Fig. 1C displays responses anchored in the “habitat dimension.” Boldfaced type illustrates concepts common across men and women. Italicized type illustrates concepts participants related to human-wildlife conflict (HWC). Men and women viewed some risks to local livelihoods differently. For example, within the “people” dimension, women offered breadth of detail about “overarching risk themes” and “theme attributes” and men offered depth of detail when describing “overarching risk themes” and “theme attributes.” Even when men and women similarly identified the “overarching risk themes” of flooding, deforestation, and agriculture within the “habitat” dimension, the “theme attributes” and “attribute descriptions” were different.

Within the “people” dimension, women offered breadth of detail about “overarching risk themes” and “theme attributes” such as human health effects (e.g., malaria, sexually transmitted disease, alcohol), polygamy, infrastructure, and poverty. In contrast, men offered depth of detail when describing “overarching risk themes” and “theme attributes” within the “people” dimension (e.g., lack of funds for infrastructure, which specifically included roads, buildings, schools, and clinics) (Fig. 1a). Men identified a greater number of species in the “wildlife” dimension [e.g., buffalos (*Syncerus caffer*), crocodiles, elephants, hippopotamus (*Hippopotamus amphibius*), hyenas, lions, porcupines (*Hystrix africaeaustralis*)] as posing risks to livelihoods than did women [e.g., buffalos, elephants, hyenas, lions, porcupines]. Only women identified wildlife scarcity and disease transmission as “overarching risk themes” within the “wildlife” dimension. One woman noted, “Kudu [*Tragelaphus strepsiceros*] are becoming few [sic] (Participant # 24).” Only men identified competition between wildlife and livestock (Fig. 1b). One man stated that competition between livestock and wildlife results because “livestock use the same source of resources (e.g., water) [as wildlife][sic](Participant # 42).” Men and women similarly identified the “overarching risk themes” of flooding, deforestation, and agriculture within the “habitat” dimension. However, the “theme attributes” and “attribute descriptions” for these concepts were different. Women noted the connection between flooding and poverty whereby men noted the connection between flooding and infrastructure (e.g., roads, buildings, villages) agriculture, life and livestock (Fig. 1c).

### Participants' wildlife concept maps

**Figure 2:**
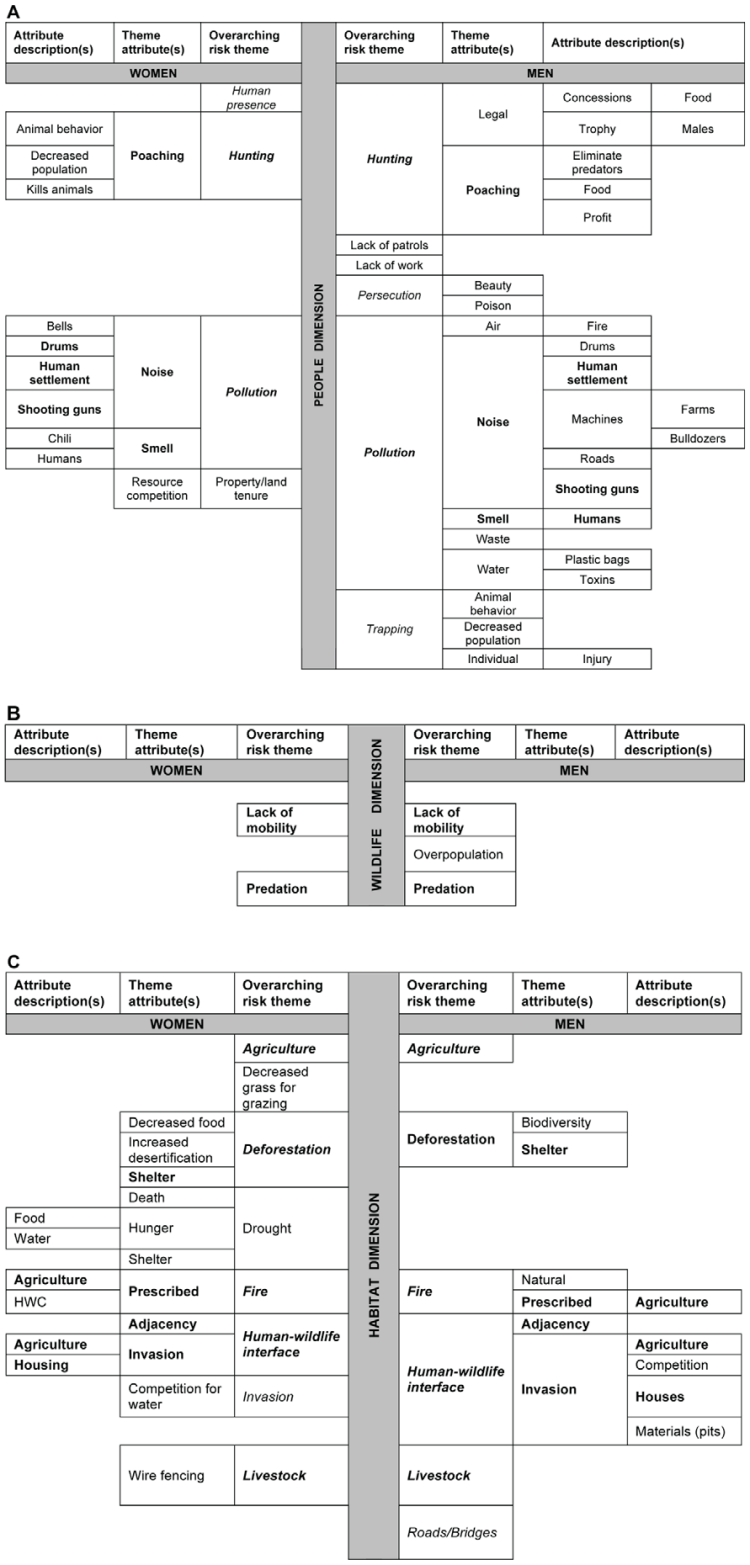
Concept maps illustrating participants' perceptions of risk to wildlife. Focus group participants (n = 32; men = 15; women = 17) generated a list of risks to local wildlife and described them. Responses are displayed in a gender-segregated concept map presenting “overarching risk themes,” “theme attributes.” and “attribute descriptions.” Fig. 2A displays responses anchored in the “people dimension,” Fig. 2B displays responses anchored in the “wildlife dimension,” and Fig. 2C displays responses anchored in the “habitat dimension.” Boldfaced type illustrates concepts common across men and women. Italicized type illustrates concepts participants related to human-wildlife conflict (HWC). Men and women identified diverse “overarching risk themes,” theme attributes” and “theme descriptions.” For example, although men and women identified pollution, including noise, as a threat to wildlife, the attribute noise was “described” differently. Men and women identified “overarching risk themes” of human activities such as hunting; only men distinguished legal from illegal hunting and noted how lack of work created risks.

Men and women identified diverse “overarching risk themes” to wildlife. Only women noted land tenure and only men noted a lack of work as being “overarching risk themes” to wildlife within the “people” dimension. “Theme attributes” and “theme descriptions” varied as well. Although men and women identified pollution, including noise, as a threat to wildlife, the attribute noise was described differently. Women defined noise as including drums, human settlement, and shooting guns whereas men defined noise as including drums, human settlement, shooting guns, machines and roads. Only men distinguished the “theme attribute” of air and water pollution as a threat to wildlife, while only women described “smell pollution” as consisting of both humans and chili used for wildlife deterrence. Men and women identified “overarching risk themes” of human activities such as hunting; however only men distinguished legal and illegal hunting and also noted how lack of work and patrols created risks (Fig. 2a). Both men and women similarly defined predation as an “overarching risk theme” within the “wildlife” dimension. Men and women similarly conceptualized the “overarching risk themes” of wildlife mobility and predation (Fig. 2b).

Men and women similarly described many “overarching risk themes” within the “habitat” dimension, for example agriculture, deforestation, the human-wildlife interface, and fire (Fig. 2c). One man noted, “…veld [bush] fire destroys the habitats of wildlife and this activity is mainly caused by human beings [sic] (Participant # 71).” Also within this dimension, women identified “overarching risk themes” related to non-human activities such as drought. For example, one woman noted, “This [drought] can make animals not live near people because of poor living [as] the area will be without water and grass [sic] (Participant # 73).”

### Interviews

**Figure 3:**
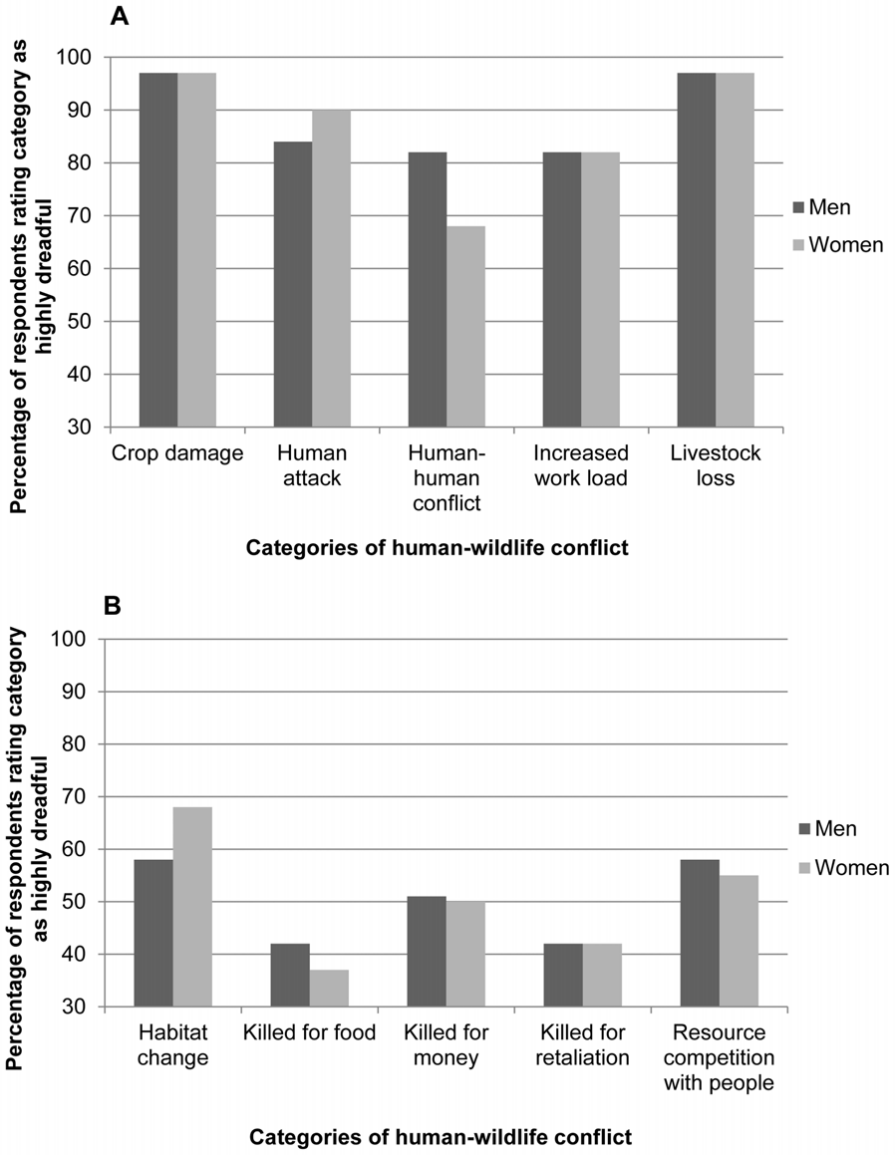
Percentage of respondents rating categories of human-wildlife conflict as highly dreadful to livelihoods and wildlife. Interview respondents (n = 76; men = 38; women = 38) rated various categories of human-wildlife conflict as being a “no,” “low,” “medium,” or “high” level of dreadfulness to them personally. Response categories were recoded into dichotomous responses of “low” and “high” (highly) dreadfulness. Fig. 3A illustrates responses when the human-wildlife conflict (HWC) risk target (i.e., the recipient of negative consequences of the risk) was local livelihoods. Fig. 3B illustrates responses when the HWC risk target was local wildlife. There was a high level of agreement among men and women when the risk target was local livelihoods, with the exception of “human-human conflict” that results from HWC, and less agreement among men and women when the risk target was local wildlife.

Interview results illustrate how study men and women differed in their perceptions of risk associated with HWC. Seventy-six individuals participated in interviews (men = 38; women = 38). Participants ranged in formal educational attainment from no school to college educated, age (18–88 years), and all participated in some form of subsistence based activity or rural industry. Study men and women reported similar risk perceptions associated with HWC with the exception of ‘worry’ associated with HWC effects on local livelihoods. Women (92.1%, *n* = 35) were more likely than men (65.8%, *n* = 25) to be highly worried (χ^2^ = 7.92, *df* = 1, *n* = 75, *P*<0.05; Table 2). There was little difference in the proportion of men and women who rated various categories of HWC-related risks to livelihoods as being highly dreadful (Fig. 3a). There was less agreement among groups regarding categories of HWC-related risks to wildlife as being highly dreadful (Fig. 3b).

**Table 2:** Effect of gender (men = 38; women = 38) on attitudes toward human wildlife conflict related risks in East Caprivi, Namibia.

	Male	Female	κ^2^ Analysis		
	% Positive or High Responses		Value	*df*	*P-value*
Risk to people from HWC^a^ acceptable	26.3	39.5	1.49	1	0.22
Risk to wildlife from HWC acceptable^a^	65.8	55.3	0.88	1	0.35
Frequency of HWC in community^b^	76.3	84.2	0.75	1	0.39
Level of control over experiencing HWC^b^	47.4	52.6	0.21	1	0.65
Worry about HWC effects on people^b^	65.8	92.1	7.92*	1	0.01
Worry about HWC effects on wildlife^b^	64.9	57.9	0.38	1	0.54

* Significant at p<0.05.

a Response options included yes or no.

b Response options included high or low.

## Discussion

Results indicate that opportunities exist to move beyond thinking about gender as an explanatory variable for understanding how different groups think about conservation issues such as managing risks associated with HWC. Knowledge about gender can be leveraged to explicitly craft and assess interventions that more purposefully respond to the needs and perceptions of different groups. Divergent risk perceptions affirm that risks need not always be experienced or considered in a uniform manner by men and women. Feminist political ecologists (e.g., Rocheleau [56]) have suggested that gender is an important variable in shaping interactions between people and their environment. Environmental knowledge is gendered and impacts, among other things, individuals' willingness to participate in environmental interventions. Thus, gendered differences in risk perceptions associated with HWC are essential to identify as part of an intervention because gender will influence willingness to participate. Fortunately, the literature provides us with compelling information about ways to engage and respond to differences in how men and women think about conservation issues. Knowing your audience is essential to effective conservation communication planning, implementation, and evaluation [57]. For example, considering gendered differences in stakeholders' interest in wildlife can be important to designing messages and delivery strategies, especially when information is presented about the nature of problems, impacts of management techniques, or reasons why certain alternatives were chosen in lieu of others [19]. Project aims are often articulated and yet mean different things to different people involved in the policy process [58]. Depending on communication goals (e.g., wildlife-focused risks or livelihood-focused risks), messages may or may not have to be formatted, delivered, and assessed differently for men and women. Measuring gendered risk perceptions (and changes over time) can reduce uncertainty for decision makers tasked with such communication or intervention activities. Decision makers can attend to differences when communicating to residents about HWC and be aware that different message content would not wholly be salient to both men and women. Such differences can also be used as criteria for accurately evaluating HWC-related interventions based on perceptions that matter to men and women.

Gendered differences in risk perceptions may signal different priorities and incentives to participate [59] in efforts to resolve HWC-related risks. A practical implication of this finding is that if local HWC interventions are framed in terms of male-oriented perceptions (e.g., lack of work), some women may not engage because they believe such efforts are irrelevant. This would be unfortunate as data indicate that HWC was clearly salient to both men and women. Environmental justice scholars (e.g., Verchick [39]) argued that failing to engage groups who are affected by and have the potential to affect environmental risks, such as HWC, would not only represent a missed opportunity for more effective risk resolution, but also an abuse of power and degree of negligence on behalf of decision makers tasked with managing risks.

Results indicate that among study participants, women's risk perceptions associated with HWC cannot be explained in isolation from men's risk perceptions. In articulating threats to livelihoods and wildlife, study participants revealed many shared: (1) goals and interests (e.g., risks to wildlife held in common such as hunting and fire); (2) superordinate goals, or objectives above the issue at hand (e.g., education, lack of food); and (3) perceptions about the salience of HWC. These similarities challenge gender essentialist assumptions (i.e., women are closer to nature than men and have natural sympathies and connections to nature whereas men do not) made by some conservationists (e.g., Jackson [59]) and may be leveraged to build common ground around wildlife conservation policies [60]. The broader implication for conservation from this finding is that although shared goals and interests may seem to be an obvious reason for cooperative wildlife management, it is not always obvious that management goals are shared. Gore et al. [61] noted the utility of applying knowledge of shared perceptions in HWC-related risk communication programs to facilitate an open flow of information, set agendas for interpersonal discussion about risks, reduce ambiguity between stakeholders, and foster more effective decision making.

Study men and women conveyed very similar conceptions of risks within the “wildlife” domain as well as perceptions about various categories of HWC. These similarities in perceptions, across groups as well as across threats to wildlife and livelihoods, may be useful fodder for HWC interventions. For example, both men and women may agree about the need for effective wildlife deterrence interventions such as chili “bombs” or “fences” to surround and protect crops from elephants. However, only women identified chili “bombs” and “fences” as being a source of risk for wildlife. The smell of chili (*capsaicin*) is believed to repel elephants and thus is promoted in a variety of elephant deterrents such as bombs, fences, and ropes that create barriers around crop fields [62]. This finding is important because women in many regions of the world, including our case study site, are responsible for much of the agricultural work beyond land clearing and plowing [21]. If women perceive risks to wildlife from chili-related interventions to be high, the implementation of a useful technique may be compromised unless risk perceptions are directly attended to as part of the intervention. Specifically, interventions should address high perceptions of risk.

The multiple congruent risks between men and women detected in our study, such as pollution, agriculture, fire, wildlife's lack of mobility, and crop damage, provoke thought about opportunities for improved risk management in general and HWC management in particular. Even though groups may have different goals regarding HWC management, it is not unreasonable for a single intervention to concomitantly manage both. For example, women mentioned disease transmission from wildlife whereas men identified competition with livestock for habitat as risks to livelihoods. Interventions or policies that aim to reduce disease transmission and habitat competition with livestock might include those that promote fencing or modified animal husbandry practices. HWC interventions may also incorporate the common goals held, but different benefits realized, by men and women. For example, among our study participants, reducing risks to livelihoods could influence risk perception among women about disease, livestock health, or poverty. For men, minimizing risks to livelihoods may influence risk perceptions about hunting and conflict among farmers. Implementing HWC-related interventions purposefully designed to target multiple goals and provide multiple gendered benefits may offer decision makers new opportunities to increase intervention potency and sustainability through evaluation that directly accounts for indicators of success. Data indicate stakeholders' risk perceptions associated with HWC are connected to and contingent upon a wide range of risks to livelihoods and wildlife. Given the financial and social resources some HWC interventions require (e.g., compensation schemes that pay landowners for wildlife-incurred property damage or loss), evaluating the social viability of interventions [6] before they are implemented could be invaluable to decision makers with limited resources. If decision makers are able to more aptly respond to local stakeholders there is the potential to increase stakeholders' perceived legitimacy of conservation interventions [63]. For example, only women identified wildlife deterrence activities such as chili “fences” as posing risks to wildlife (i.e., women characterized chili “fences” as being a form of smell pollution). In this instance, gendered risk perception could influence the social acceptability of wildlife deterrence measures such as chili “fences.” Treves et al. [6] discussed further the strong relationship between perceptions and social acceptability of HWC-related interventions. Because gender has the potential to influence perceptions it is an important response variable to consider vis à vis acceptability.

HWCs are likely to continue to pose conservation challenges in the foreseeable future. Policies, activities, and actions that effectively reduce risks to human and wildlife health and safety associated with HWC using participatory methods are currently and will likely remain in high demand. The complexity of HWC requires a conservation toolbox that offers breadth and depth of detail for building capacity to understand human relationships with wildlife, including those that are gendered. Interventions focused on human behavior (e.g., communication designed to reinforce, restrain, or maintain human behaviors) are common [1]. We know that systematic and interdisciplinary studies that adapt social science methodologies and consider local risk perceptions are irreplaceable components of effective HWC management and mitigation and promote both conservation and livelihood security [3]. Gendered risk perceptions have yet to be fully integrated into the context of participatory approaches to resolving HWC-related risks and are especially salient in conservation contexts with distinct gender roles related to interactions with wildlife. This research highlights insights that conservationists may glean from when considering gendered perceptions of HWC-related risk.

## References

[1] Gore ML, Knuth BA, Scherer CW, Curtis PD (2008). Evaluating a conservation investment designed to reduce human-wildlife conflict.. Conservation Letters.

[2] Gore ML, Knuth BA (2009). Mass media effect on the operating environment of a wildlife-related risk communication campaign.. Journal of Wildlife Management.

[3] Treves A, Wallace RB, Naughton-Treves L, Morales A (2006). Co-managing human-wildlife conflicts: a review.. Human Dimensions of Wildlife.

[4] Gore ML, Knuth BA, Curtis PD, Shanahan JE (2006). Education programs for reducing American black bear-human conflict: indicators of success?. Ursus.

[5] Ogra MV (2008). Human-wildlife conflict and gender in protected area borderlands: a case study of costs, perceptions, and vulnerabilities from Uttarakhand (Uttaranchal), India.. Geoforum.

[6] Treves A, Wallace R, White S (2009). Participatory Planning of Interventions to Mitigate Human-Wildlife Conflicts.. Conservation Biology.

[7] Hill CM (2004). Farmers' perspectives of conflict at the wildlife-agriculture boundary: some lessons learned from African subsistence farmers.. Human Dimensions of Wildlife.

[8] Woodroffe R, Thirgood S, Rabinowitz A (2005). People and wildlife: conflict or coexistence.

[9] Treves A, Karanth KU (2003). Human-carnivore conflict and perspectives on carnivore management worldwide.. Conservation Biology.

[10] Agrawal B (2001). Participatory exclusions, community forestry, and gender: an analysis for South Asia and a conceptual framework.. World Development.

[11] Raik DB, Lauber TB, Decker DJ, Brown TL (2005). Managing community controversy in suburban wildlife management: adopting practices that address value differences.. Human Dimensions of Wildlife.

[12] Naughton-Treves L (1999). Whose animals? A history of proprietary rights to wildlife in Toro, western Uganda.. Land Degradation and Development.

[13] Wilson RS (2008). Balancing emotion and cognition: a case for decision aiding in conservation efforts.. Conservation Biology.

[14] Agrawal B (2000). Conceptualizing environmental collective action: why gender matters.. Cambridge Journal of Economics.

[15] Agrawal A, Gibson C (2001). Communities and the environment: ethnicity, gender, and the state in community-based conservation.

[16] Lauber TB, Knuth BA (2000). Suburban residents criteria for evaluating contraception and other deer management techniques.. Human Dimensions of Wildlife.

[17] Zanetell BA (2001). Consensus-Based Collaboration in Watershed Management: Quixotic Notion or the Environmental Pot of Gold?.

[18] Anthony ML, Knuth BA, Lauber TB (2004). Gender and citizen participation in wildlife management decision-making.. Society and Natural Resources.

[19] Lauber TB, Anthony ML, Knuth BA (2001). Gender and ethical judgments about suburban deer management.. Society and Natural Resources.

[20] Gore ML, Knuth BA, Curtis PD, Shanahan JE (2006). Stakeholder perceptions of risk associated with human-black bear conflicts in New York's Adirondack Park campgrounds: Implications for theory and practice.. Wildlife Society Bulletin.

[21] Hunter ML, Hitchcock RK, Wyckoff-Baird B (1990). Women and wildlife in Southern Africa.. Conservation Biology.

[22] United Nations (2010). The Millennium Development Goals Report: United Nations Department of Economic and Social Affairs..

[23] Ray I (2007). Women, water, and development.. Annual Review of Environment and Resources.

[24] Nightingale A (2006). The nature of gender: work, gender, and environment.. Environment and Planning: Society and Space.

[25] Faasen H, Watts S (2007). Local community reaction to the ‘no take’ policy on fishing in the Tsitsikamma National Park, South Africa.. Ecological Economics.

[26] Martin D (2008). Gender and urban perceptions of nature and protected areas in Bañados del Este Biosphere Reserve.. Environmental Management.

[27] Baral N, Jensen JT (2007). Decentralization and people's participation in conservation: a comparative study from the Western Terai landscape of Nepal.. The International Journal of Sustainable Development and World Ecology.

[28] Loyd KAT, Miller CA (2010). Influence of demographics, experience, and value orientations on preferences for lethal management of feral cats.. Human Dimensions of Wildlife.

[29] Kuehn DM, Dawson CP, Hoffman R (2006). Exploring fishing socialization among male and female anglers in New York's Eastern Lake Ontario area.. Human Dimensions of Wildlife.

[30] Jacobson CA, Brown TL, Decker DJ (2007). Gender-biased data in survey research regarding wildlife.. Society and Natural Resources.

[31] Kellert SR, Berry JK (1987). Attitudes, knowledge, and behaviors toward wildlife as affected by gender.. Wildlife Society Bulletin.

[32] Zinn H, Pierce C (2002). Values, gender, and concern about potentially dangerous wildlife.. Environment and Behavior.

[33] Gilligan C (1982). In a different voice: psychological theory and women's development.

[34] Ogra MV (2009). Attitudes toward resolution of human-wildlife conflict among forest-dependent agriculturalists near Rajaji National Park, India.. Human Ecology.

[35] Slovic P (1987). Perception of risk.. Science.

[36] Gore ML, Wilson RS, Siemer WF, Wieczorek Hudenko H, Clarke CE (2009). Application of risk concepts to wildlife management: special issue introduction.. Human Dimensions of Wildlife.

[37] Flynn J, Slovic P, Mertz CK (1994). Gender, race, and perception of environmental health risks.. Risk Analysis.

[38] Gustafson PE (1998). Gender differences in risk perception: theoretical and methodological perspectives.. Risk Analysis.

[39] Verchick RRM (2004). Feminist theory and environmental justice.. New perspectives on environmental justice: gender, sexuality and activism.

[40] Cutter SL, Tiefenbacher JI, Solecki WD (1992). En-gendered fears: femininity and technical risk perception.. Individual Crisis Quarterly.

[41] O'Connell-Rodwell CE, Rodwell T, Rice M, Hart LA (2000). Living with the modern conservation paradigm: can agricultural communities co-exist with elephants? A five-year case study in East Caprivi, Namibia.. Biological Conservation.

[42] Jones BTB, Barnes JI (2006). Human wildlife conflict study: Namibian case study..

[43] Bernard HR (2006). Research methods in anthropology: qualitative and quantitative approaches.

[44] Trochim WMK (2001). The research methods knowledge base.

[45] Kahler JS (2010). Local Perceptions of Risk and Vulnerability Associated with Human-Wildlife Conflicts in Namibian Conservancies.. Retrieved from ProQuest Digital Dissertations.

[46] Smith K, Barrett CB, Box PW (2000). Participatory risk mapping for targeting research and assistance: an example from East African pastoralists.. World Development.

[47] Morgan DL (1993). Successful focus groups.

[48] Reid J (1990). The Dirty Laundry of ESL Survey Research.. TESOL Quarterly.

[49] Roster C, Albaum G, Rogers R (2006). Can cross-national/cultural studies presume etic equivalency in respondent's use of extreme categories of Likert rating scales?. International Journal of Market Research.

[50] Chachamovich E, Fleck MP, Power M (2009). Literacy affected ability to adequately discriminate among categories in multipoint Likert Scales.. Journal of Clinical Epidemiology.

[51] Glaser B, Strauss A (1967). The discovery of grounded theory-strategies for qualitative research.

[52] Miles MB, Huberman AM (1994). Qualitative data analysis: an expanded sourcebook.

[53] LeCompte MD, Goetz JP (1983). Playing with ideas: analysis of qualitative data..

[54] Conover M (2002). Resolving human-wildlife conflicts.

[55] SPSS Inc (2010). PASW Statistics 18.0..

[56] Rocheleau DE (2008). Political ecology in the key of policy: from chains of explanation to webs of relation.. Geoforum.

[57] Shanahan JE, Gore ML (2012). Communication for human dimensions of wildlife..

[58] Harrison E (1997). Fish, feminists, and the FAO: translating ‘gender’ through different institutions in the development process.. Getting institutions right for women in development.

[59] Jackson C (1993). Doing what comes naturally? Women and environment in development.. World Development.

[60] Wondolleck JM, Yaffee YM (2000). Making collaboration work: lessons from innovation in natural resource management..

[61] Gore ML, Knuth BA, Curtis PD, Shanahan JE (2007). Campground managers and user perceptions of risk associated with negative human-black bear interactions.. Human Dimensions of Wildlife.

[62] Hedges S, Giumaryadi D (2010). Reducing human-elephant conflict: do chilies help deter elephants from entering crop fields?. Oryx.

[63] Kuperan K, Sutinen JG (1998). Blue water crime: deterrence, legitimacy, and compliance in fisheries.. Law & Society Review.

[64] Namibian Association of CBNRM Support Organizations (NACSO) (2009). Namibia's communal conservancies: a review of progress 2008.

